# Efficient gene transfer into zebra finch germline-competent stem cells using an adenoviral vector system

**DOI:** 10.1038/s41598-021-94229-x

**Published:** 2021-07-20

**Authors:** Kyung Min Jung, Young Min Kim, Jin Lee Kim, Jae Yong Han

**Affiliations:** Department of Agricultural Biotechnology and Research Institute of Agriculture and Life Sciences, College of Agriculture and Life Sciences, Seoul National University, 1 Gwanak-ro, Gwanak-gu, Seoul, 08826 Korea

**Keywords:** Biotechnology, Cell biology, Developmental biology, Genetics, Molecular biology, Stem cells, Zoology

## Abstract

Zebra finch is a representative animal model for studying the molecular basis of human disorders of vocal development and communication. Accordingly, various functional studies of zebra finch have knocked down or introduced foreign genes in vivo; however, their germline transmission efficiency is remarkably low. The primordial germ cell (PGC)-mediated method is preferred for avian transgenic studies; however, use of this method is restricted in zebra finch due to the lack of an efficient gene transfer method for the germline. To target primary germ cells that are difficult to transfect and manipulate, an adenovirus-mediated gene transfer system with high efficiency in a wide range of cell types may be useful. Here, we isolated and characterized two types of primary germline-competent stem cells, PGCs and spermatogonial stem cells (SSCs), from embryonic and adult reproductive tissues of zebra finch and demonstrated that genes were most efficiently transferred into these cells using an adenovirus-mediated system. This system was successfully used to generate gene-edited PGCs in vitro. These results are expected to improve transgenic zebra finch production.

## Introduction

Zebra finch (*Taeniopygia guttata*), a representative songbird, is a useful model animal for investigating the brain, behavioral, and neurobiological processes because it can learn vocalizations by imitating a singing adult, similar to humans who acquire spoken language. This trait is not observed in traditional model organisms such as rodents and non-human primates and is rarely found in other mammals^1,2^. In zebra finch, functional gene analyses have been performed by controlling gene expression via transient knockdown or overexpression of genes of interest in vivo^3–6^. Transgenic models that express the human mutant huntingtin gene or mutant forms of cAMP response element-binding protein have been developed by injecting lentiviruses containing transgenes into blastoderms of fertilized eggs to target primordial germ cells (PGCs). However, the low efficiency of this method indicates that improvements are required for studies of transgenic songbirds^2,7,8^.

Germline-competent stem cells, including PGCs and spermatogonial stem cells (SSCs), are animal cells that can transfer all genetic information to the next generation, self-renew, and differentiate into mature gametes. These characteristics make germline-competent stem cells a suitable resource for generating transgenic animals. PGCs, which give rise to sperm and oocytes, are the most actively studied germline-competent stem cells in avian species, especially chickens, and have become a very popular system for creating a variety of transgenics related to bioreactors and disease models^9–13^. SSCs, the other type of male germline-competent stem cells in adult testes that are precursors in spermatogenesis, have been successfully isolated from mammalian species, cultured, and used for transgenic production^14,15^. SSCs of several bird species including chickens, pheasants, and quails have been isolated and cultured, but practical applications for producing transgenic birds using these cells are limited^16–20^. In zebra finch, although studies have reported the isolation and manipulation of PGCs, reports of exogenous genes being efficiently delivered into these cells are limited^21,22^. Recently, transgenic zebra finches were generated using PGCs and lentiviral vectors, but the green fluorescent protein (GFP) transgene transferred by lentiviruses was lowly expressed and only detected using an anti-GFP antibody in sectioned tissues of transgenic finches^22^. Thus, further investigations of PGCs and SSCs are required to facilitate in vitro manipulation of these cells and obtain basic data in order to induce genome modifications in zebra finch germline-competent stem cells.

A reliable method to obtain germline-competent zebra finch cell lines has not been developed. In vivo transfection and gene transfer into primary cells are transgenic technologies that do not require established cell lines, but it is difficult to achieve high efficiency with these methods. Virus-mediated transduction may be an alternative for gene transfer into isolated primary germline-competent stem cells, which have low transfection rates using universal transfection methods such as lipofection. Viral vectors have been used to transfer genes into cells and living organisms. Adenoviruses are an important type of viral vector that have a relatively high transduction efficiency in target cells and infect a large range of host cells, including dividing and nondividing cells and several types of stem cells that are difficult to transfect^23^. Adenovirus-mediated gene transfer into mammalian germline stem cells has been successfully performed in vivo and in vitro^23,24^. Targeted gene disruption using adenovirally delivered Clustered Regularly Interspaced Short Palindromic Repeats (CRISPR)/Cas9 was also recently reported in birds^25,26^.

In this study, we describe an efficient method to transfer genes into primary germline-competent stem cells, including PGCs and SSCs, of zebra finch that overcomes the aforementioned limitations. We additionally induced a targeted genome modification in zebra finch PGCs using the established gene transfer method and CRISPR/Cas9 system. The biological functions of the genome-edited cells were verified by their incorporation into host reproductive tissues upon in vivo transplantation. These results could facilitate the production of transgenic and genetically modified zebra finches in future research.

## Results

### Culture and characterization of zebra finch PGCs

**Figure 1:**
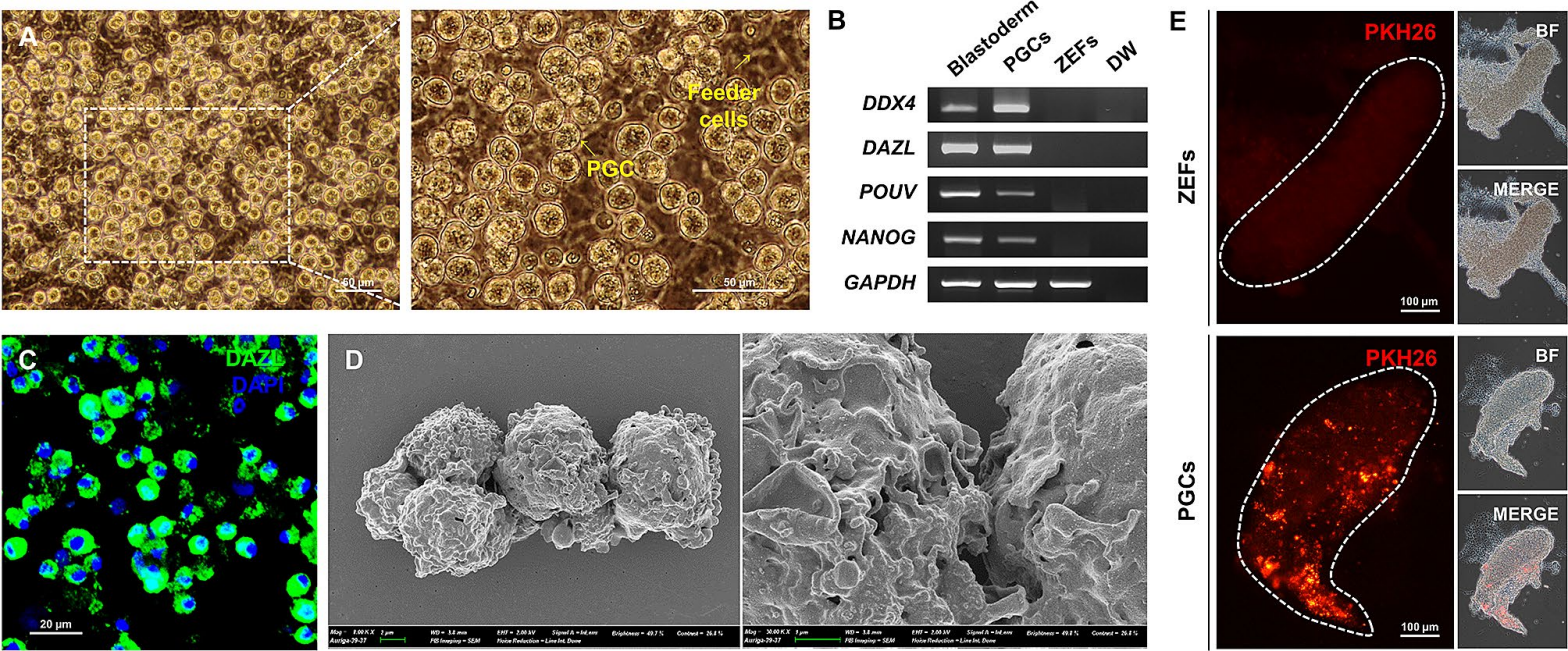
Isolation and characterization of zebra finch PGCs. (**A**) Morphology of zebra finch PGCs cultured for a short duration in vitro. (**B**) RT-PCR analysis of zebra finch PGCs. Blastoderm was used as a positive control and primary ZEFs were used as a negative control. DW, distilled water. (**C**) Zebra finch PGCs were identified by immunostaining with an anti-DAZL antibody. (**D**) Scanning electron microscopy of zebra finch PGCs. (**E**) Migration of PGCs in recipient zebra finch embryos. Approximately 500 PGCs or ZEFs were labeled with PKH26 red fluorescent dye and injected separately into the dorsal aorta of zebra finch embryos at HH13–16. Fluorescent cells were observed in recipient embryonic gonads at HH28.

The whole gonadal cells were isolated from zebra finch embryos at Hamburger–Hamilton (HH) stage 28 and cultured for a short duration in vitro, and then suspended cells, which were presumed to be PGCs, were harvested and characterized (Fig. 1A). Expression of germ cell marker genes (*DDX4* and *DAZL*) and pluripotency marker genes (*POUV* and *NANOG*), which are regarded as representative PGC markers in avian species, was detected by RT-PCR analysis (Fig. 1B and Supplementary Fig. 1). Immunostaining with an anti-DAZL antibody showed that these cells were positive for DAZL (Fig. 1C). Scanning electron microscopy showed that these cells had an irregular morphology with abundant microvilli-like structures (Fig. 1D). Additionally, to characterize the migration of these cells and their ability to incorporate into the embryonic gonads via the bloodstream, approximately 500 cells that had been cultured for 15–20 days were labeled with PKH26 red fluorescent dye and immediately injected into the bloodstream of zebra finch embryos at HH13–16. After 3 days (at HH28), labeled cells were successfully incorporated into the recipient embryonic gonads (mean number of migrated cells per gonad, 182.7 ± 17.2) (Fig. 1E). These results demonstrate that PGCs are obtained by short-term culture, and the PGCs culture was dependent on individual embryos and was not sex-specific (Supplementary Fig. 2).

### Isolation and characterization of zebra finch SSCs

**Figure 2:**
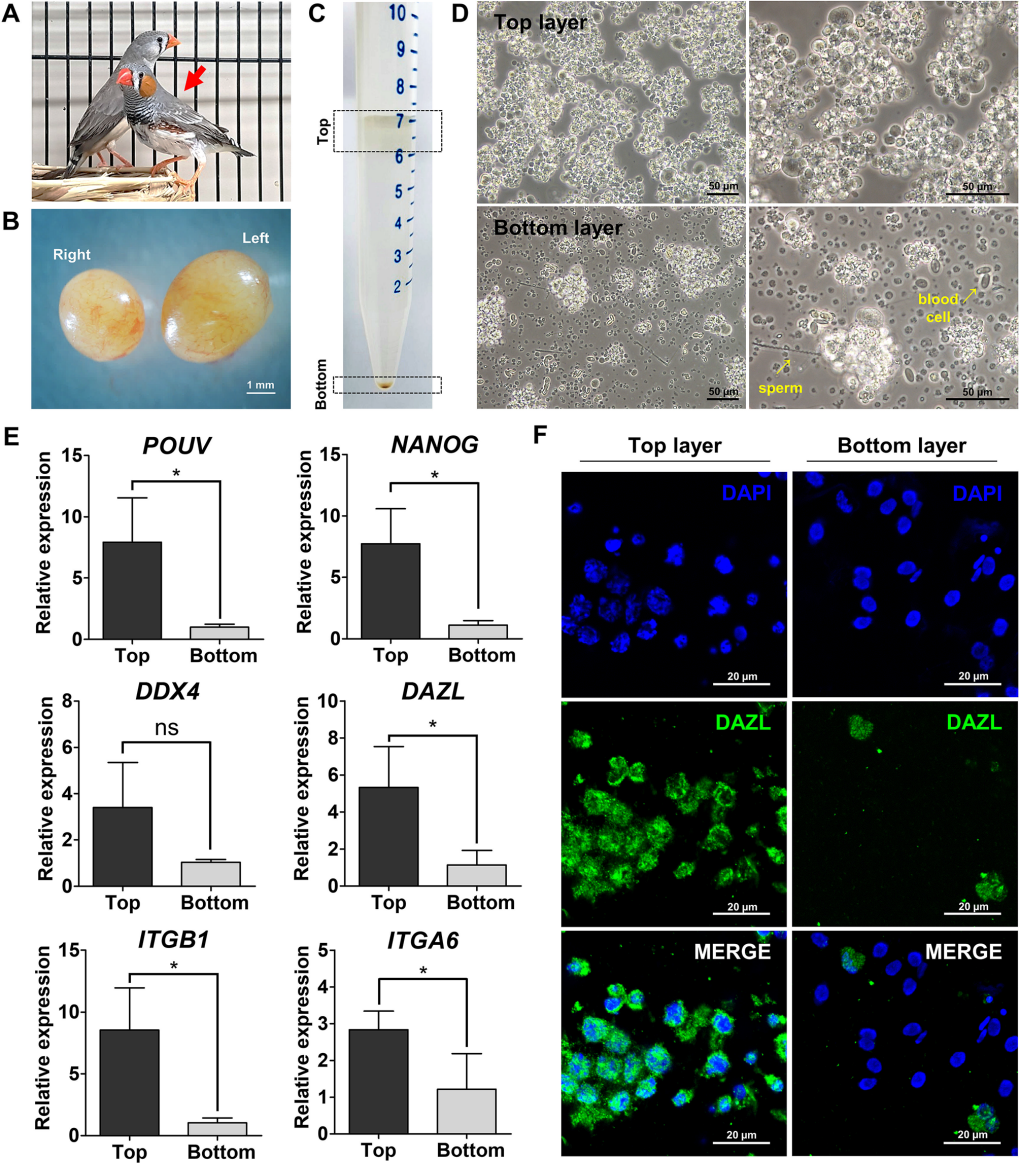
Enrichment and characterization of zebra finch SSCs. (**A**) Adult female and male zebra finches. The zebra finch indicated by the red arrow has an orange cheek patch, which is indicative of males. (**B**) Isolated testes of an adult male zebra finch. (**C**,**D**) Isolation of testicular germ cells by Ficoll density gradient centrifugation. Testicular cells were separated into two layers (dotted boxes). The top and bottom layers were selected and plated in culture plates. (**E**) Gene expression analysis of the two layers of testicular germ cells cultured for 1 day. Quantitative RT-PCR analysis of six distinct and well-accepted pluripotency marker genes (*POUV* and *NANOG*), germ cell marker genes (*DDX4* and *DAZL*), and SSC marker genes (*ITGB1* and *ITGA6*). Significant differences between the top and bottom layers are shown (Student’s t-test; *p < 0.05, ns = not significant). (**F**) Cells in the top and bottom layers were immunostained with an anti-DAZL antibody.

SSCs, an important germline-competent stem cell resource for generating transgenic animals, were isolated from testes of an adult male zebra finch (Fig. 2A,B) by the Ficoll density gradient centrifugation method, which successfully enriches SSCs from quails without a specific marker^20^. Testicular cells were separated into two main layers, named the top and bottom layers (Fig. 2C). Each layer was plated on a 6-well culture plate. The top layer contained cells that were round, had clear cytoplasm, and were of distinct sizes (20–25 µm in diameter) in comparison with those in the bottom layer and thus these cells were morphologically similar to SSCs (Fig. 2D)^27^. Quantitative RT-PCR assessing expression of six distinct and well-accepted pluripotency marker genes (*NANOG* and *POUV*), germ cell marker genes (*DDX4* and *DAZL*), and SSC marker genes (*ITGB1* and *ITGA6*) was performed to examine enrichment of SSCs in the top layer. Expression of all tested genes except for *DDX4*, a germ cell marker, was significantly higher in cells from the top layer than in cells from the bottom layer (Fig. 2E). Immunohistochemistry showed that there were more DAZL-positive cells in the top layer than in the bottom layer (Fig. 2F). These results suggest that SSCs from zebra finch can be successfully enriched without using specific markers.

### Validation of gene transfer efficiencies into zebra finch PGCs

**Figure 3:**
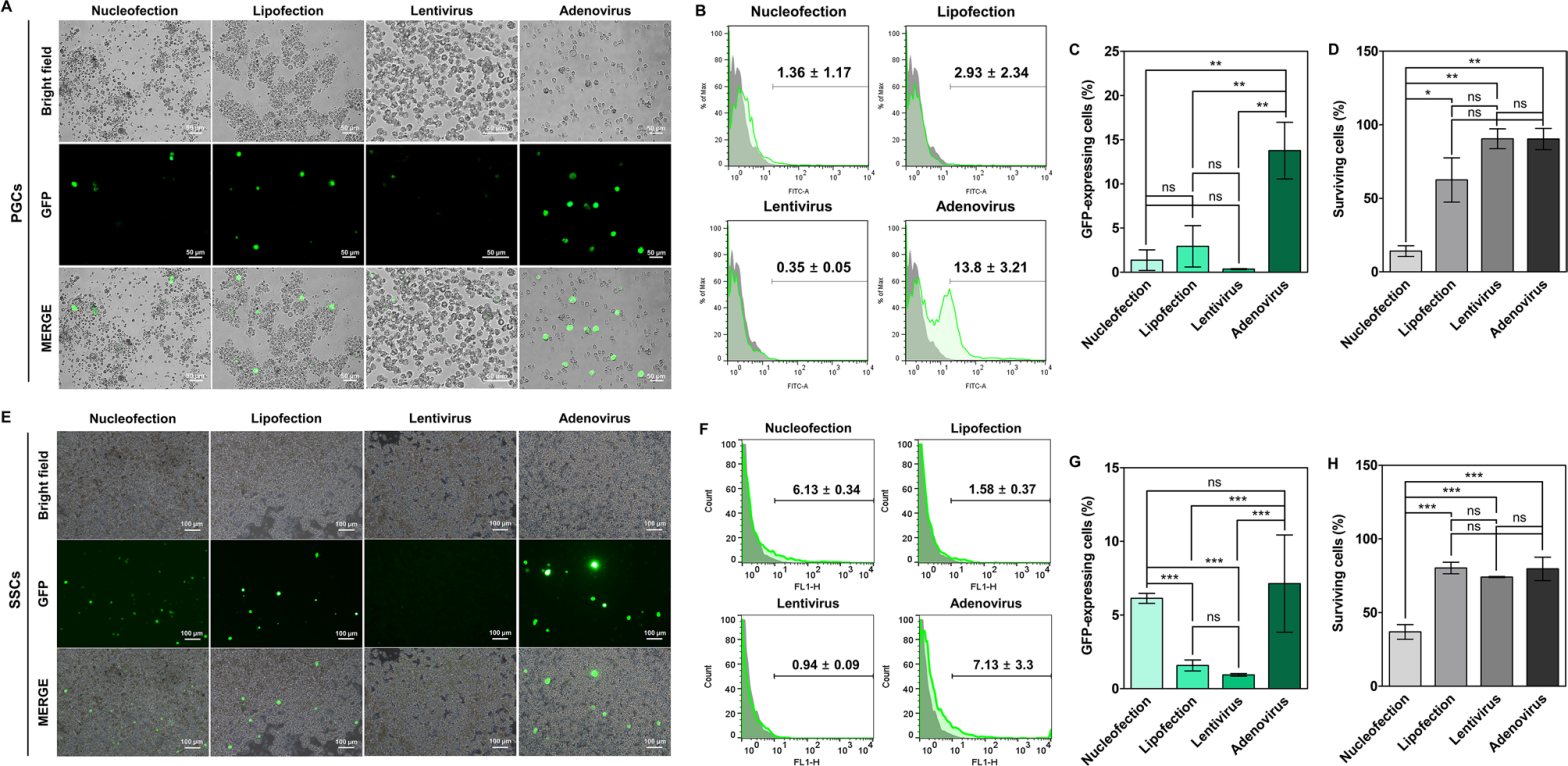
Comparison of the gene transfer efficiencies of various transfection methods in zebra finch germline-competent stem cells in vitro. (**A**–**D**) Gene transfer efficiency in zebra finch PGCs. The GFP gene was transferred into zebra finch PGCs using nucleofection, lipofection, a lentivirus, and an adenovirus. (**A**) Representative morphology of transfected zebra finch PGCs. (**B**,**C**) Transfection efficiencies measured by flow cytometry. Significant differences between the four groups are shown (one-way ANOVA; **p < 0.05; ns = not significant). (**D**) The percentage of surviving cells was determined by trypan blue staining. Significant differences between the four groups are shown (one-way ANOVA; *p < 0.05; **p < 0.005; ns = not significant). (**E**–**H**) Gene transfer efficiency in zebra finch SSCs. (**E**) Representative morphology of transfected zebra finch SSCs. (**F**,**G**) Transfection efficiencies measured by flow cytometry. Significant differences between the four groups are shown (one-way ANOVA; ***p < 0.0001; ns = not significant). (**H**) The percentage of surviving cells was determined by trypan blue staining. Significant differences between the four groups are shown (one-way ANOVA; ***p < 0.0001; ns = not significant).

Foreign genes need to be introduced into zebra finch PGCs with high efficiency. Therefore, a variety of transfection methods were applied and comparatively analyzed. Zebra finch PGCs were transfected with the GFP expression vector via nucleofection or lipofection. Also, zebra finch PGCs were transduced with a lentiviral or adenoviral vector containing the GFP coding sequence. Flow cytometry revealed that the percentage of GFP-positive PGCs was low following nucleofection-, lipofection-, and lentivirus-mediated transfection (1.36 ± 1.17%, 2.93 ± 2.34%, and < 1%, respectively), and was highest following adenovirus-mediated transfection (13.8 ± 3.21%) (Fig. 3A–C). The percentage of viable cells was significantly lower in the nucleofection group than in the other groups (Fig. 3D). Therefore, the adenoviral vector system, which was highly efficient compared with the other tested transfection methods and did not affect cell viability, was regarded as the most suitable method for gene transfer into zebra finch gonadal PGCs in our experimental conditions.

### Validation of gene transfer efficiencies into zebra finch SSCs

Transfection of zebra finch SSCs has not been attempted; therefore, it was necessary to test different transfection methods and determine which was best. Thus, a variety of transfection methods were applied and comparatively analyzed. Before the comparative experiment, three nucleofection programs (A-023, L-029, and X-001) were tested and the optimal one was selected (Supplementary Fig. 3). The transduction efficiency was highest with the X-001 program, and cell viability did not differ between the three nucleofection programs (Supplementary Fig. 3). Zebra finch SSCs were transfected with the GFP expression vector using electroporation-based nucleofection or liposome-based lipofection. Also, zebra finch SSCs were transduced with a lentiviral or adenoviral vector containing the GFP coding sequence. The percentage of GFP-positive cells was determined by flow cytometry (Fig. 3E,F). The percentage of GFP-positive cells was low following lipofection- and lentivirus-mediated transfection (1.58 ± 0.37% and 0.94 ± 0.09%, respectively) and relatively high following nucleofection- and adenovirus-mediated transduction (6.13 ± 0.34% and 7.13 ± 3.3%, respectively) (Fig. 3E–G). The percentage of viable cells was significantly lower in the nucleofection group than in the other groups (Fig. 3H). Thus, we concluded that the adenoviral vector system is optimal for delivering exogenous genes into zebra finch SSCs without affecting cell viability.

### Adenoviral CRISPR/Cas9-mediated targeted mutagenesis in zebra finch germline-competent stem cells

**Figure 4:**
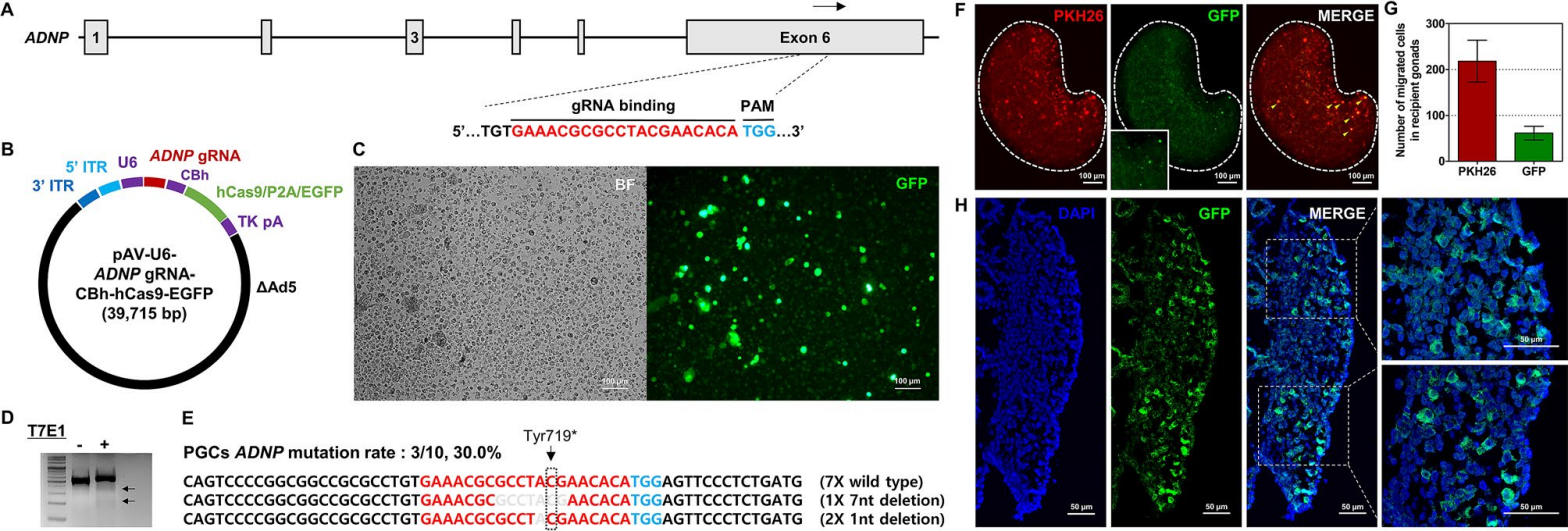
CRISPR/Cas9-mediated gene targeting using the adenovirus-mediated method in zebra finch PGCs. (**A**) Schematic of the gRNA targeting the zebra finch *ADNP* gene. The gRNA sequence located in exon 6 is shown in red letters and the PAM sequence is shown in light blue letters. (**B**) Construction of the CRISPR/Cas9 adenoviral vector containing the selected gRNA sequence targeting the *ADNP* gene. (**C**) CRISPR/Cas9 adenovirus-transduced zebra finch PGCs. GFP expression indicates transduced cells. (**D**,**E**) Knockout efficiency in zebra finch PGCs according to the T7E1 assay and DNA sequencing. Total PCR products were inserted into the T-vector and analyzed by DNA sequencing. Red letters indicate gRNA recognition sequences, light blue letters indicate PAM sequences, and gray letters indicate deletions. The dotted box indicates the Tyr719* residue located in exon 6 of the *ADNP* gene, a major point mutation causing ADNP syndrome. (**F**,**G**) In vivo migration of genome-modified zebra finch PGCs in recipient embryos. Approximately 1000 cells transduced with the adenovirus carrying the CRISPR/Cas9 system were labeled with PKH26 red fluorescent dye, injected into the dorsal aorta of zebra finch embryos at HH13–16, and incubated until HH28. Fluorescent cells were observed in recipient gonads and counted. (**H**) Some recipient gonads were paraffin-sectioned and immunostained with an anti-GFP antibody.

Gene editing was attempted using the adenoviral gene transfer method in vitro to verify the potential of zebra finch germline-competent stem cells for transgenic applications. Activity-dependent neuroprotective protein (ADNP) syndrome is an autistic-like disorder caused by mutations of the human *ADNP* gene. The *ADNP* gene is robustly expressed in the cerebrum of young male zebra finches, corroborating with singing behavior and the development of the cerebral song system. Thus, we selected *ADNP* as the target gene to verify the genome-editing potential of the adenoviral method in zebra finch PGCs. The gRNA sequence was designed to target the Tyr719* residue located in exon 6 of the *ADNP* gene, one of the major point mutations that cause ADNP syndrome (Fig. 4A and Supplementary Fig. 4). CRISPR/Cas9 plasmids encoding the specific gRNA (pX459/*ADNP* gRNA) were generated (Supplementary Fig. 4B) and their ability to disrupt the target gene was tested in primary zebra finch fibroblasts (ZEFs). Sequencing analysis confirmed that the designed gRNA sequence induced nucleotide deletions at the targeted locus with an efficiency of 34.5% (10/29) in primary ZEFs (Supplementary Fig. 4E). For targeted knockout of the *ADNP* gene in zebra finch PGCs, adenoviruses carrying hCas9 and the gRNA sequence were generated and applied to PGCs in vitro (Fig. 4B). Transient GFP signals due to delivery of the GFP coding sequence by the adenoviral vector were detected in zebra finch PGCs 4 days after transduction (Fig. 4C). Nucleotide modifications of the targeted locus were detected by the T7E1 assay (Fig. 4D and Supplementary Fig. 5). Sequencing analysis detected nucleotide deletions at the gRNA-targeted locus of the *ADNP* gene with an efficiency of 30% (3/10) in zebra finch PGCs (Fig. 4E). To evaluate the off-target effects in *ADNP* edited PGCs, we analyzed six putative off-target sites by the TA cloning method, and we detected no off-target mutation (Supplementary Fig. 6). Same approach was also attempted with zebra finch SSCs, but mutagenesis of the target gene was not observed (data not shown). These results demonstrate that the adenovirally delivered CRISPR/Cas9 system can induce nucleotide deletions at targeted gene loci in zebra finch PGCs, germline-competent stem cells, but the method must be improved for SSCs.

### In vivo incorporation of genome-edited zebra finch PGCs

To investigate whether zebra finch PGCs maintain their in vivo migration properties after genome-modification using the adenoviral system, genome-modified PGCs were transplanted in vivo. Approximately 1000 PGCs subjected to adenoviral transduction (~ 30% of which were genome-edited with GFP signals) were labeled with PKH26 and immediately injected into the bloodstream of recipient zebra finch embryos at HH13–16. After 3 days, at HH28, GFP-expressing PGCs were successfully incorporated into recipient embryonic gonads (Fig. 4F,G). Of the 1000 injected cells, 218 ± 46 cells were incorporated into recipient gonads (PKH26-labeled cells in both gonads were counted) and 28.1 ± 6.9% of them (61 ± 15 cells) expressed GFP (PKH26-labeled and GFP-expressing cells were counted). For detailed observation of migrated cells, some recipient embryonic gonads containing migrated cells were paraffin-sectioned and immunostained with an anti-GFP antibody. GFP-expressing cells were observed in sectioned recipient embryonic gonads (Fig. 4H). These data suggest that targeted genome-modified zebra finch PGCs maintain their germ cell properties in vivo and can be applied to create transgenic embryos.

## Discussion

Zebra finch is an ideal organism for studying the basis of neurogenesis and speech disorders in humans, but transgenic studies are lacking, and the germline transmission efficiency is very low^1,2,7,8^. Generation of germline chimeras using germline-competent stem cells, such as PGCs and SSCs, is regarded as an efficient approach to produce transgenic animals of avian species. It is difficult to apply mammalian transgenic systems such as embryo transfer and nuclear transfer methods in avian species due to their oviparous characteristics^9,28^. The PGC-mediated system is the most prominent method to produce germline transgenic models of avian species^29,30^. A few studies reported PGC-mediated production of transgenic zebra finches. Gessara et al. recently attempted PGC-mediated production of transgenic zebra finches using a lentivirus system. However, improvements are required and gene transfer methods other than the lentivirus-mediated approach must be developed. Moreover, gene editing technologies have not been applied to zebra finch, indicating many unexplored areas related to zebra finch transgenics^22^. Therefore, in this study, we isolated and characterized two types of primary germline-competent stem cells from zebra finch, established an efficient gene transfer system by performing comparative experiments, and reported targeted gene editing in zebra finch for the first time using the selected method.

We investigated the efficiency with which exogenous DNA was introduced into zebra finch PGCs using nucleofection, lipofection, a lentivirus, and an adenovirus. The efficiency of the lentivirus-mediated method was the lowest, which differs from previous results of introducing EGFP with high efficiency by lentivirus system. Gessara et al., used circulating PGCs, not gonadal PGCs, and explained that the high efficiency is due to the expression of the low-density lipoprotein receptor (LDLR) family gene of PGC, which was increased when cultured for 10 days in their culture conditions, suggesting that the origin of PGC and culture conditions affect the transfection efficiency^2,7,8^. Lipid-DNA complex-mediated transfection is effective in chicken PGCs^30,31^, but not in some primary cell types^32,33^. This limitation is believed to be primarily due to intracellular barriers, including poor endocytosis, endosome escape, and nuclear localization of the transformed DNA^34^. Nucleofection, an electroporation technique, can easily transfer exogenous genes into the cytoplasm and nucleus of some stem cells and primary cells that are difficult to transfect^35,36^, but was ineffective in this study and reduced the viability of zebra finch PGCs. In zebra finch SSCs, the nucleofection- and adenovirus-mediated methods had similar efficiencies, but nucleofection reduced cell viability and thus the adenovirus-mediated method was selected as the optimal approach. However, the efficiency of the adenovirus-mediated method in SSCs greatly varied between experiments and must be improved.

In zebra finch PGCs, targeted gene editing using the adenoviral CRISPR/Cas9 system was successful and had an efficiency of ~ 30%. This efficiency may be improved by introducing a gRNA sequence with a better editing efficiency in the future. Transduced zebra finch PGCs could be amplified by establishing a long-term culture system for these cells and subsequent in vitro selection. Genome-editing was also attempted in zebra finch SSCs but failed. Gene delivery using adenoviruses results in transient expression of the transgene; therefore, the efficiency estimated according to GFP expression is not proportional to the editing efficiency^37^. Further optimization, such as introduction of polymers including polybrene, poly-l-lysine, and polyethyleneimine that enhance the adenoviral transduction efficiency, is required to increase the efficiency of gene transfer and improve gene editing in zebra finch SSCs^38–40^.

Adenoviruses are versatile tools for gene transfer and expression that can be applied in vitro to obtain gene-edited cells as a resource for generating transgenics and can also be directly used to target PGCs in vivo. In quail, an adenovirus was directly injected into the blastoderm containing PGCs, leading to the generation of transgenic quails with a high germline transmission efficiency^25^. Thus, it is expected that the adenovirus system, which had the highest efficiency in zebra finch germline-competent stem cells, can be applied directly in vivo as well as in vitro, thereby expanding research concerning the production of transgenic zebra finches.

In summary, we isolated and characterized primary germline-competent stem cells, PGCs and SSCs, from zebra finch, optimized the gene transfer method, and confirmed that the adenovirus-mediated method was most efficient in both cell types. Delivery of the CRISPR/Cas9 system to PGCs using adenoviruses enabled gene targeting in zebra finch for the first time. This study is an important foundation for the production of transgenic zebra finches.

## Methods

### Experimental animals

The care and experimental use of zebra finches were approved by the Institute of Laboratory Animal Resources, Seoul National University, Korea. All procedures, including zebra finch maintenance, reproduction, and sample collection, were governed by standard operating protocols according to a standard management program at the University Animal Farm, and the Animal Genetic Engineering Laboratory, Seoul National University.

### Ethics statement

All experimental procedures and care of zebra finches were approved by the Institute of Laboratory Animal Resources, Seoul National University. All methods were performed in accordance with ARRIVE (Animal Research: Reporting of In Vivo Experiments) guidelines and approved by the Institutional Animal Care and Use Committee (IACUC, SNU-200305-2-1) of Seoul National University.

### Culture of zebra finch PGCs

Gonads retrieved from zebra finch embryos at HH 28 were enzymatically digested and then cultured in vitro using a previously reported method^21^. Briefly, gonadal cells were suspended in PGC culture medium, which comprised knockout Dulbecco’s Modified Eagle Medium (DMEM; Thermo Fisher Scientific, Waltham, MA, USA), supplemented with 10% fetal bovine serum (Hyclone, Logan, UT, USA), 2% chicken serum (Millipore Sigma, Burlington, MA, USA), 1 × nucleosides (Millipore Sigma), 2 mM l-glutamine (Thermo Fisher Scientific), 1 × nonessential amino acids, β-mercaptoethanol, 10 mM sodium pyruvate, 1 × antibiotic–antimycotic reagents (Thermo Fisher Scientific), and 10 ng/mL human basic fibroblast growth factor (Millipore Sigma). The PGCs-enriched suspension was sub-cultured every 4–5 days for about 1 month, and a fraction of adherent cells was used as a feeder layer.

### Sexing PCR

Sexing of embryos was performed by PCR amplification of the chromodomain-helicase-DNA-binding protein *(CHD)1* gene using genomic DNA extracted from extraembryonic tissues and gene-specific primers (Supplementary Table 1)^41^.

### Density gradient centrifugation

Density gradient centrifugation of testicular cells was performed using a previously reported method with some modifications^20^. Testes were surgically dissected from sexually mature zebra finches (6 months old) and dissociated with 0.05% trypsin–EDTA. The cell suspension was filtered through a 40 µm nylon cell strainer. Then, 4 mL of the cell suspension in phosphate-buffered saline (PBS) was mixed with 3 mL of Ficoll-Paque PLUS solution (GE Healthcare Life Science, Chicago, IL, USA) and centrifuged at 800 × *g* for 30 min. After centrifugation, cells from differently layered fractions (top and bottom layers) were harvested and washed three times with PBS containing antibiotic–antimycotic reagents. Cells were pelleted and resuspended in culture medium, which comprised DMEM supplemented with 10% fetal bovine serum, 1 × nonessential amino acids, 15 ng/mL glial cell-derived neurotrophic factor (PeproTech, Rocky Hill, NJ, USA), 10 ng/mL basic fibroblast growth factor, 0.55 mM β-mercaptoethanol, 2 mM l-glutamine, 1 µM sodium pyruvate, and 1 × antibiotic–antimycotic solution at 37 °C in a humidified CO_2_ incubator containing 5% CO_2_. Cells were used for characterization or transfection at 1 day after seeding, and the transfected cells were maintained up to 4 days in vitro.

### Immunocytochemical analysis of zebra finch PGCs and SSCs

Zebra finch PGCs and SSCs were dried on glass slides, fixed in 4% paraformaldehyde for 10 min, washed three times with PBS, and permeabilized with 0.1% Triton X-100 for 10 min. After three washes with PBS, cells were blocked with blocking buffer (PBS containing 5% goat serum and 1% bovine serum albumin) for 1 h and then incubated with an anti-DAZL antibody^42^ at 4 °C overnight. After three washes with PBS, cells were incubated with an Alexa Fluor 488-conjugated secondary antibody for 1 h at room temperature. Cells were finally mounted using ProLong Gold antifade reagent with DAPI and analyzed under a confocal fluorescence microscope (Carl Zeiss GmbH, Jena, Germany).

### RT-PCR and quantitative RT-PCR

Total RNA samples were prepared using TRIzol reagent (Thermo Fisher Scientific) and reverse-transcribed into cDNAs using a SuperScript III Reverse Transcription Kit (Thermo Fisher Scientific) according to the manufacturer’s protocol. cDNAs were amplified by PCR using primer sets for the predicted zebra finch DEAD-box helicase 4 (*DDX4*), deleted in azoospermia-like (*DAZL*), POU domain class 5 transcription factor 3 (*POUV*), Nanog homeobox (*NANOG*), and glyceraldehyde 3-phosphate dehydrogenase (*GAPDH*) genes. PCR reactions comprised 35 cycles at 95 °C for 30 s, 60 °C for 30 s, and 72 °C for 1 min. Gene expression levels were measured using EvaGreen dye (Biotium, Hayward, CA, USA) and a CFX96 Real-Time PCR Detection System (Bio-Rad, Hercules, CA, USA). Relative gene expression was quantified using the following formula: 2^−ΔΔCt^, where ΔΔCt = (Ct of the target gene − Ct of *GAPDH*)_top layer_ − (Ct of the target gene − Ct of *GAPDH*)_bottom layer_. The primer sets used for RT-PCR and quantitative RT-PCR are listed in Supplementary Table 2.

### Scanning electron microscopy

PGCs were fixed in 2% glutaraldehyde and postfixed in 1% osmium tetroxide at 4 °C for 2 h. After dehydration in a graded series of increasing concentrations of ethanol, the samples were immersed in hexamethyldisilazane and then dried. The samples were coated with gold palladium using a Leica EM ACE200 low-vacuum coater (Leica Microsystems, Buffalo Grove, IL, USA) and observed using a Sigma field emission scanning electron microscope (Carl Zeiss GmbH).

### Migration assay of zebra finch PGCs

About 500 PGCs were injected into the dorsal aorta of zebra finch embryo at HH 13–16. The eggs were sealed with medical-grade silicone adhesive (Kwik-Cast, World Precision Instruments, Sarasota, FL, USA) and further incubated until HH 28. Fluorescent cells in recipient embryonic gonads were detected.

### Transfection of PGCs and SSCs

A total of 5 × 10^5^ PGCs cultured for 15–20 days or 1 × 10^6^ SSCs cultured for 1 day after isolation were transfected with 1–2 μg of the GFP expression vector in a 12-well culture plate using an Amaxa Nucleofector (V buffer and X-001 program) or Lipofectamine 2000 reagent (Invitrogen). The nucleofection program was selected by testing three nucleofection programs (A-023, L-029, and X-001) using SSCs. PGCs and SSCs were also transduced with lentivirus (1 × 10^9^ VP/mL) or adenovirus (1 × 10^10^ VP/mL) purchased from Vigene Bioscience (Vigene Bioscience, Rockville, MD, USA) at a multiplicity of infection (MOI) of 100. Cell viability was calculated as the number of viable cells divided by the total number of cells within the grid on the hemacytometer using trypan blue staining (Millipore Sigma). Transfected or transduced cells were resuspended in PBS and analyzed with a FACSCalibur system (BD Biosciences, San Jose, CA, USA). Subsequent analyses were performed using FlowJo software (Treestar, Ashland, OR, USA).

### Primary culture of zebra finch embryonic fibroblasts (ZEFs)

The primary culture of ZEFs was prepared from the muscles of 6-day-old zebra finch embryos^43^. Several embryos (n = 3–5) were extracted from fertilized eggs, their head, limbs, and organs were removed, and the remaining contents were minced. Single-cell populations were obtained by 0.05% trypsin–EDTA treatment and maintained in DMEM (Hyclone) containing 10% fetal bovine serum (Hyclone) and 1 × antibiotic–antimycotic reagents (Thermo Fisher Scientific). Cells were seeded at a density of 5 × 10^5^ cells/well in a 12-well culture plate and grown at 37℃ in an incubator containing 5% CO_2_.

### Plasmid construction

The CRISPR/Cas9 vector targeting the *ADNP* gene was constructed using the pX459 vector, as previously reported^44^. The gRNA sequences targeting *ADNP* gene was designed by Geneious prime software considering on-target score (Supplementary Fig. 4C). To insert the gRNA sequence into the CRISPR/Cas9 vector, sense and antisense oligonucleotides were synthesized (Bionics, Seoul, South Korea) (Supplementary Table 3) and annealed using the following thermocycling conditions: 30 s at 95 °C, 2 min at 72 °C, 2 min at 37 °C, and 2 min at 25 °C. The annealed oligonucleotides were ligated into the pX459 vector using the Golden Gate assembly method, and the constructed CRISPR/Cas9 vectors were validated by Sanger sequencing. Adenoviruses carrying hCas9 and *ADNP* gRNA sequences were produced and purified by ViGene Biosciences according to their standard protocol (CsCl gradient purification and plaque assay) and the vector designated as pAV-U6-gRNA-CBh-hCas9-EGFP.

### Transfection and genomic DNA sequencing of ZEFs

To validate the mutation efficiency of the designed gRNA sequence targeting the *ADNP* gene, dissociated ZEFs (1 × 10^6^) were resuspended in nucleofection solution containing 10 μg of CRISPR/Cas9 plasmids and nucleofection was performed using the X-001 program. Genomic DNA was extracted from transfected cells. Genomic regions encompassing the CRISPR/Cas9 target sites were amplified using specific primer sets (Supplementary Table 3). The PCR amplicons were annealed into the pGEM-T Easy Vector and sequenced by Sanger sequencing (Bionics). The sequencing results were analyzed using Geneious Prime software.

### Genome-editing of zebra finch PGCs in vitro

A total of 5 × 10^5^ PGCs cultured for 15–20 days were transduced with the adenovirus produced by Vigene Bioscience at a MOI of 100. Adenovirus for genome-editing was produced by transfecting 293A cells with a designed plasmid (pAV-U6-gRNA-CBh-hCas9-EGFP) by lipofection, and then purified by iodixanol gradient ultracentrifugation and tittered by IHC-immunohistochemistry (Vigene Bioscience). Genomic DNA was extracted from the cells 4 days after transduction. Genomic regions encompassing the CRISPR/Cas9 target sites were amplified using specific primer sets (Supplementary Table 3). The PCR amplicons were annealed to the pGEM-T Easy Vector and sequenced by Sanger sequencing (Bionics), and the sequencing results were analyzed using Geneious Prime software.

### Analysis of off-target mutations in edited PGCs

Potential off-target sites were screened on the basis of high homologous scores, using the CCTop online prediction tool, and the 3 closest off-target sites were selected. These off-target sites shared 15–16 matched nucleotides with up to 11 consecutive matched sequences of the 20-base pair gRNA sequences (Supplementary Fig. 6A). The PCR amplification of the potential off-target regions using genomic DNAs from edited PGCs was conducted. The PCR amplicons were annealed to the pGEM-T Easy Vector and sequenced by Sanger sequencing (Bionics), and the sequencing results were analyzed using Geneious Prime software.

### Transplantation of edited PGCs in vivo

About 1000 transduced PGCs were injected into the dorsal aorta of zebra finch embryo at HH 13–16. After injection, the eggs were sealed with medical-grade silicone adhesive (Kwik-Cast, World Precision Instruments) and further incubated until HH 28. Fluorescent cells in the recipient embryonic gonads were quantified under a fluorescence microscope.

### Immunohistochemical analysis of embryonic gonads

Embryonic gonads with mesonephric tissues of zebra finch embryos at HH 28 were paraffin-embedded and sectioned (thickness, 8–10 μm). After deparaffinization, sections were washed with PBS and blocked with blocking buffer (PBS containing 5% goat serum and 1% bovine serum albumin) for 1 h at room temperature. Sections were then incubated with a rabbit anti-GFP primary antibody (Thermo Fisher Scientific) at 4 °C overnight. After washing with PBS, sections were incubated with an Alexa Fluor 488-conjugated secondary antibody (Thermo Fisher Scientific) for 1 h at room temperature. After washing, sections were mounted using ProLong Gold antifade reagent with DAPI and visualized using a confocal fluorescence microscope (Carl Zeiss GmbH).

### Statistical analysis

Relative expression of genes was compared between the top and bottom fractions separated by Ficoll density gradient centrifugation using the Student’s t-test with GraphPad Prism statistical software (GraphPad Software, La Jolla, CA, USA). The transfection efficiency and cell viability using different transfection methods were compared by an ANOVA and Bonferroni’s Multiple Comparison Test using GraphPad Prism statistical software. All values are means ± standard deviation (n = 3).

### Approval of animal experiments

All experimental procedures and care of zebra finches were approved by the Institute of Laboratory Animal Resources, Seoul National University. All methods were performed in accordance with the guidelines and regulations of the Institutional Animal Care and Use Committee of Seoul National University (IACUC, SNU-200305-2-1). All procedures, including zebra finch maintenance, reproduction, and sample collection, were governed by standard operating protocols according to a standard management program at the University Animal Farm, Seoul National University and the Animal Genetic Engineering Laboratory, Seoul National University.

## Supplementary Information

Supplementary Information.

## Data Availability

The datasets generated during and/or analyzed during the current study can be found in the figures, tables, and supplementary information or are available upon request.
