# Supplementary material for: Efficient gene transfer into zebra finch germline-competent stem cells using an adenoviral vector system
Source: Sci Rep. 2021 Jul 20;11:14746. doi: 10.1038/s41598-021-94229-x (PMC8292312; doi:10.1038/s41598-021-94229-x)
Supplement: Supplementary file 1 — Supplementary Information. [file 41598_2021_94229_MOESM1_ESM.pdf]

Title: Efficient gene transfer into zebra finch germline-competent stem cells using an adenoviral vector system

Kyung Min Jung[1], Young Min Kim[1], Jin Lee Kim[1], and Jae Yong Han[1,*]

[1]Department of Agricultural Biotechnology, and Research Institute of Agriculture and Life Sciences, College of Agriculture and Life Sciences, Seoul National University, Seoul 08826, Korea

[*]Corresponding author: Jae Yong Han, Ph.D. Department of Agricultural Biotechnology, College of Agriculture and Life Sciences, Seoul National University, 1 Gwanak-ro, Gwanak-gu, Seoul 08826, Korea. Tel: +82-2-880-4810; FAX: +82-2-874-4811; E-mail: jaehan@snu.ac.kr

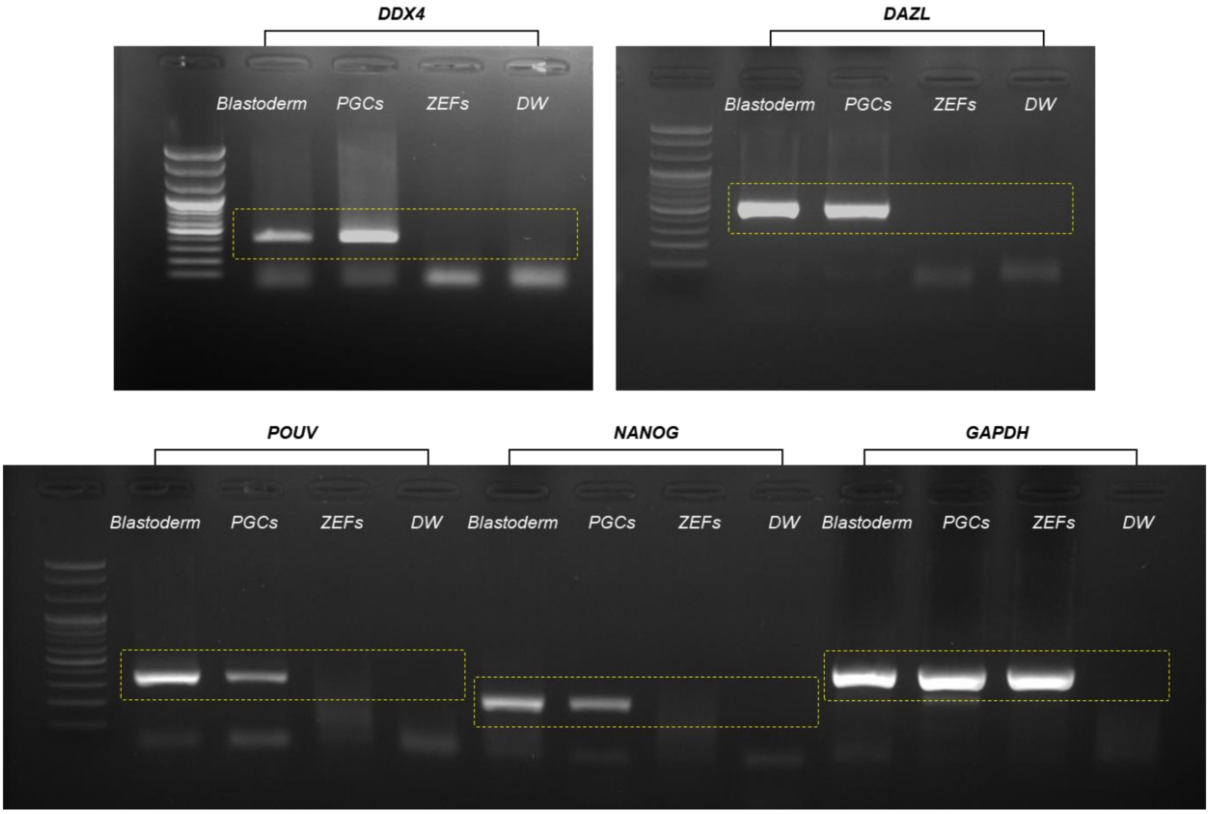

Supplementary figure 1. RT-PCR analysis of zebra finch PGCs. Expression of germ cell-marker genes (*DDX4* and *DAZL*) and pluripotency marker genes (*POUV* and *NANOG*) were analyzed in zebra finch PGCs. Blastoderm was used as a positive control and primary ZEFs were used as a negative control. ZEFs, zebra finch embryonic fibroblasts; DW, distilled water. The parts shown in Fig. 1B are indicated by yellow dashed lines.

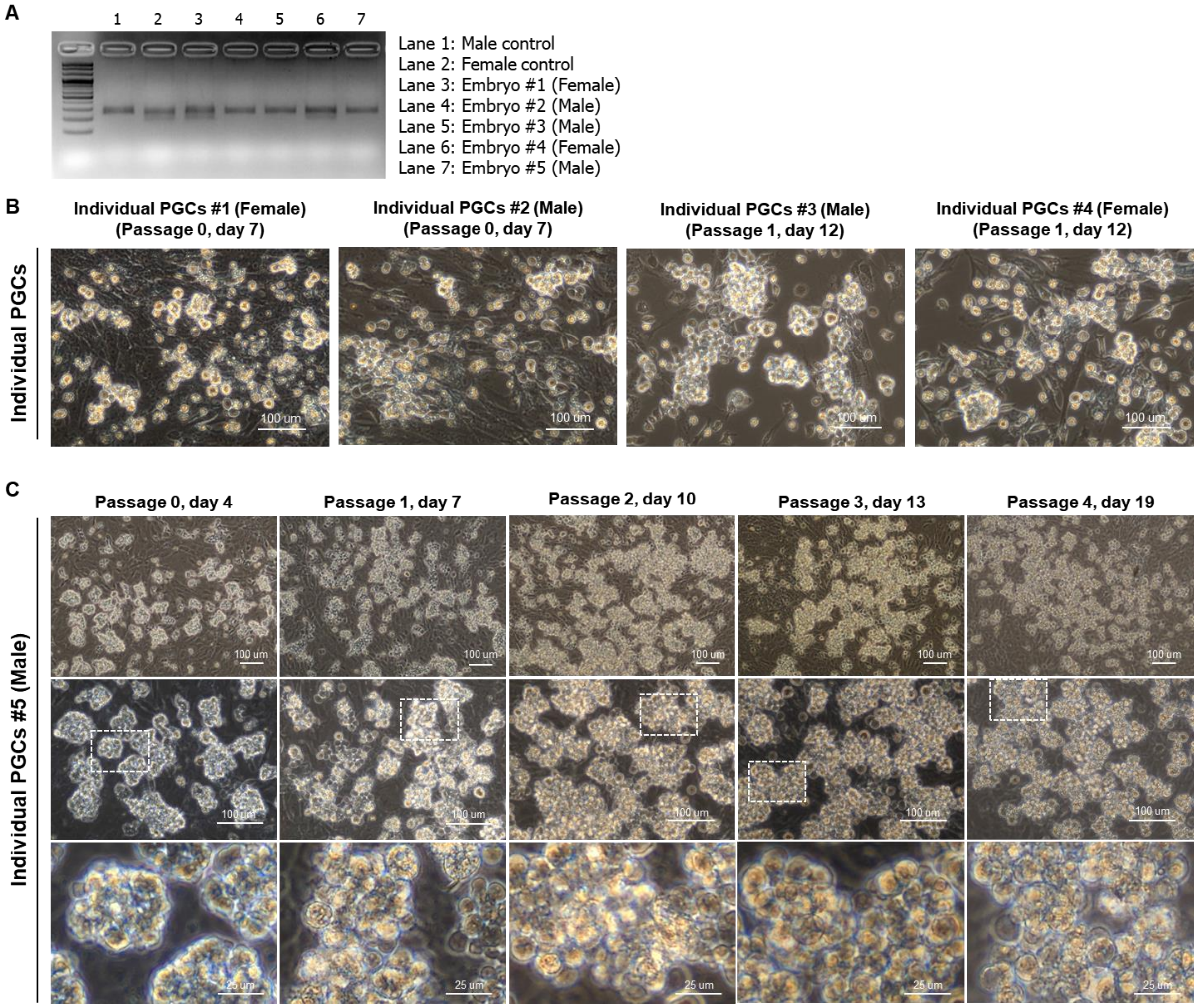

Supplementary figure 2. Individual culture of zebra finch PGCs *in vitro*. (A) Sexual identification of the embryos from which the gonad was extracted. Genomic DNA was extracted from extra embryonic tissues for sexing PCR. Sexing of the embryos was performed by chromodomain-helicase-DNA binding protein *(CHD) 1* gene PCR amplification of the genomic DNA. Samples with known sex were used as positive controls. (B-C) Individual culture of zebra finch PGCs *in vitro*. (C) Passaging status of PGCs *in vitro*. White dashed boxes indicate the location of the enlarged images at the bottom. Scale bar, 100 um and 25 um (Enlarged images).

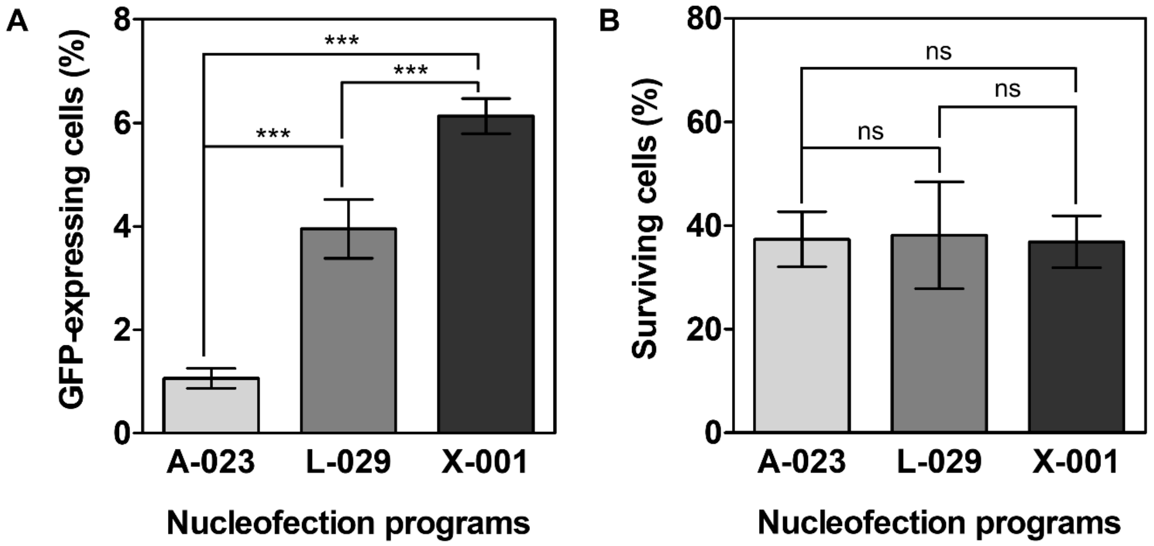

Supplementary figure 3. Selection of the optimal nucleofection program for gene transfer into zebra finch SSCs. (A) Comparison of the transfection efficiency between three nucleofection programs. A total of 1×10^6 cells was transfected using 100 µl of solution V and three nucleofection programs with an Amaxa nucleofector. GFP-expressing cells were counted by flow cytometry 4 days after transfection. (B) Viability of transfected zebra finch SSCs was analyzed by trypan blue staining 4 days after transfection. Significant differences between the three groups are shown (one-way ANOVA; ***$p < 0.001$; ns = not significant)

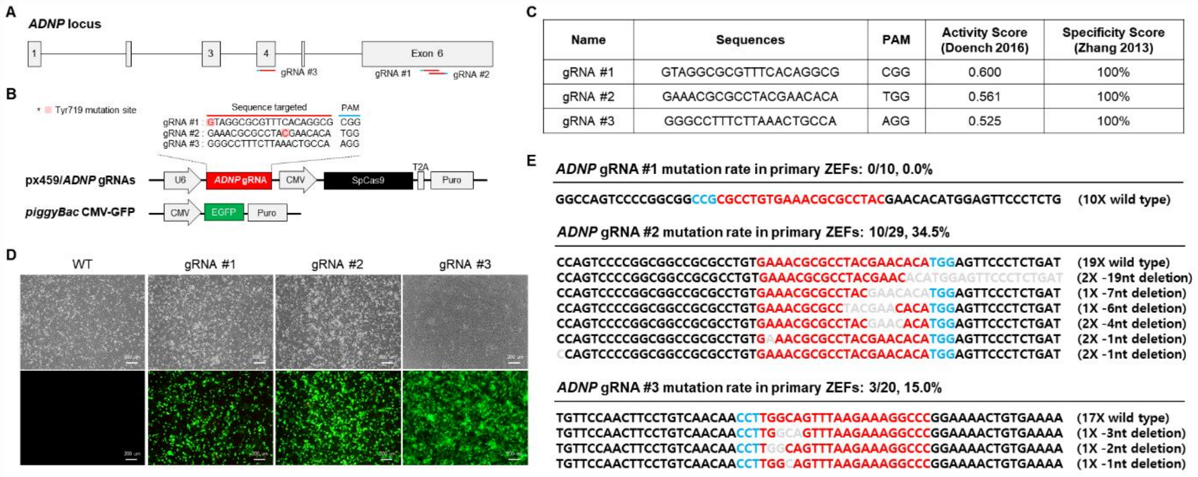

Supplementary figure 4. Verification of the efficiency with which the *ADNP*-targeting gRNA induced indels in primary ZEFs. (A) Schematic of the gRNAs used to target the zebra finch *ADNP* gene. The gRNAs are indicated by the red bars and the PAM sequences are indicated by the light blue bars. (B) Structure of the CRISPR/Cas9 plasmid targeting the *ADNP* gene. A GFP expression vector was co-transfected to transiently express GFP. (C) Information of designed gRNA sequences from Geneious prime software. (D) Images of transfected primary ZEFs. (E) DNA sequences of *ADNP* loci in transfected cells. Genomic DNA was extracted from transfected cells and PCR-amplified. PCR amplicons were inserted into the T-vector and analyzed by DNA sequencing. Red letters indicate gRNA recognition sequences, light blue letters indicate PAM sequences, and gray letters indicate deletions.

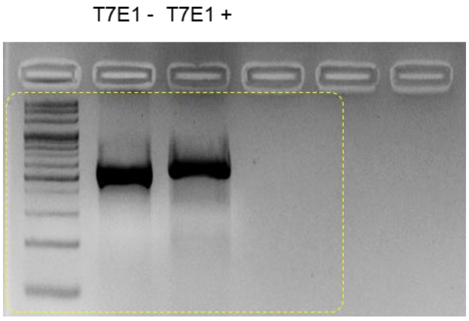

Supplementary figure 5. T7E1 assays performed on genomic DNA of zebra finch PGCs edited by adenoviral CRISPR/Cas9 system. Genomic DNA PCR products of edited PGCs were treated with T7E1. The parts shown in Fig. 4D is indicated by yellow dashed line.

A gRNA sequence: GAAACGCGCCTACGAACACA

No.	Coordinates	strand	Number of mismatches	target_seq	PAM	Frequency of off-target
1	Chr 24:3054088-3054110	-	4	GAAAGATG[CCTAGGAACACA]	TGG	0/12
2	Chr 11:3045156-3045178	-	4	GAAACACC[CCTATGAGCACA]	AGG	0/7
3	Chr 2:113893455-113893477	-	5	AACACACT[GCTACGAACACA]	GGG	0/5
4	Chr Z: 48188916-48188938	-	5	GATCCGCC[CCTAAGTACACA]	TGG	0/4
5	Chr 3: 91614082-91614104	-	5	AAAAAGCT[CCTACCACCACA]	AGG	0/4
6	Chr 12: 10151622-10151644	-	5	GTAAGGCA[CCTCTGAACACA]	GGG	0/4

B

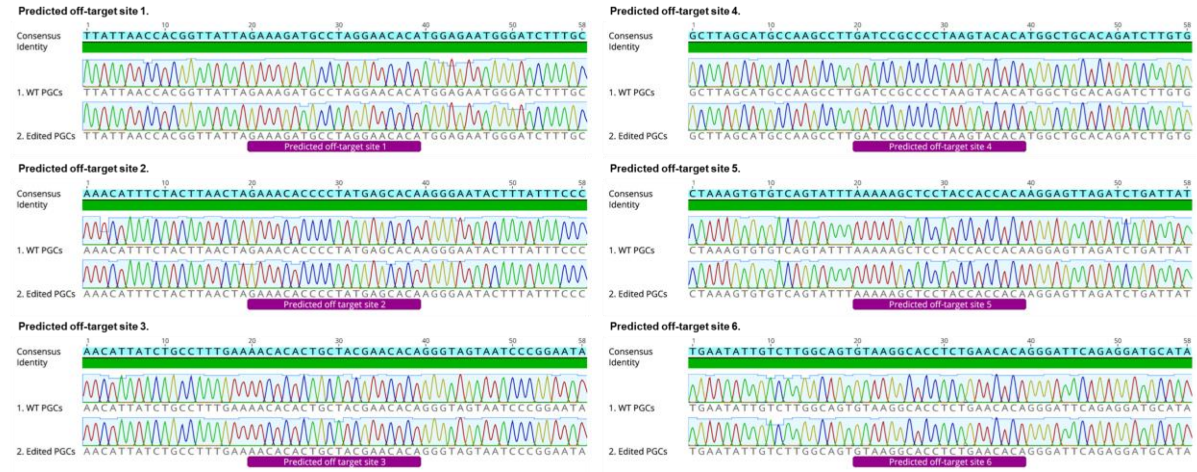

Supplementary figure 6. Investigation of potential off-target sites of *ADNP* gRNA #2. (A) Prediction of potential off-target sites by CCTop prediction tool (Off-target sites with mismatched bases less than four were not predicted). Frequency of off-target means number of off-target clones/sequenced clones. (B) Sanger sequencing chromatograms at each putative off-target region in edited PGCs.

Supplementary table 1. Primer sequences used for sexing PCR

Usage	ID	Sequence (5’→3’)	Size (bp)
Sexing PCR	W1	GGGTTTTGACTGACTAACTGATT	179
	W2	GTTCAAAGCTACATGAATAAACA	
	Z1	GTGTAGTCCGCTGCTTTTGG	242
	Z2	GTTCGTGGTCTTCCACGTTT	

Supplementary table 2. Primer sequences used for RT-PCR and quantitative RT-PCR

Usage	ID	Sequence (5'→3')	
		**Forward**	**Reverse**
RT-PCR	*POUV*	GCACAAGCGCATCATGCTGG	CCTCAGCGATCTGGGACATC
	NANOG	TCCCCAGCTTCATCCAGTTC	CACCTGCTTGTAGGTGAGCC
	DDX4	TGTGCCCAGACAGGATCAGG	GCATGCGGTCTGCTTCATCC
	DAZL	GCTGAAACTGGGGCCAGCAA	CATGCCCCTGTCCACAGACT
	GAPDH	CCATGCCATCACAGCCACAC	CCTTGGATGCCATGTGGACC
Quantitative RT-PCR	qRT-*POUV*	ATCGAATTGGGCGAACTGGT	AGGTTGTGCAAGATGACCGT
	qRT-*NANOG*	TCAAATTCCTGCGTGCAACC	CTGCTGCACGTATGTCTCCA
	qRT-*DDX4*	CTTCCGCAAGTGTGTGAAGC	AGAACCAGACCCGGACTACA
	qRT-*DAZL*	CCAGAGCACCTCTGACACTG	ATGGGGTGCCAAGGTAACTG
	qRT-*ITGB1*	AAGGAGAGCCAACATCTGCC	GCTCCCCGACTCTTAGATGC
	qRT-*ITGA6*	GGGCCTTACGAAGTTGGTGA	GCCATCACTGTTGAGGTCCA
	qRT-*GAPDH*	CCACATGGCATCCAAGGAGT	AGAGCTAAGCGGTGGTGAAC

Supplementary table 3. Oligonucleotide sequences used for genome editing

Usage	ID	Sequence (5'→3')
PX459 CRISPR/Cas9 expression vectors construction	*ADNP* gRNA #1	caccgGTAGGCGCGTTTCACAGGCG
		aaacCGCCTGTGAAACGCGCCTACc
	ADNP gRNA #2	caccgGAAACGCGCCTACGAACACA
		aaacTGTGTTCGTAGGCGCGTTTCc
	ADNP gRNA #3	caccgTCTCAATCTCTCTCCGAGTG
		aaacCACTCGGAGAGAGATTGAGAc
T7E1 assay and sequencing	*ADNP* T7E1_F	CCAGGTGATCCAGACGGTTC
	ADNP T7E1_R	GCAGCACCCCGGGTTTATAT
Off-target sequencing	Predicted off-target site 1	F: TTGTGCAGTGTCTAGCCAGG
		R: AGTTCTCTACAGGGTCCCCC
	Predicted off-target site 2	F: ACTCCTGACTCAATGCTCCAG
		R: ACACCATTTGACTTTGCATCAAGT
	Predicted off-target site 3	F: TTTTGGGCTCTCTTTCCTCAA
		R: TCCCCAAATCACTCTGCTCAC
	Predicted off-target site 4	F: ATGCACAGAGTTCTCCCAGC
		R: CATCTGCCGTCTTCTCCACA
	Predicted off-target site 5	F: GGAGGATTGTGGTCATGCCA
		R: CCTCTGAGTGTACTGGGGGA
	Predicted off-target site 6	F: TCTGCAGCACTAGCAAGGTG
		R: TCTTGGTTTGGAGTTTAGGCCT
